# Supplementary material for: Are carbon emissions trading and green financial instruments synergistic? -Comprehensive quantitative research based on content analysis
Source: PLoS One. 2024 Mar 7;19(3):e0298601. doi: 10.1371/journal.pone.0298601 (PMC10919601; doi:10.1371/journal.pone.0298601)
Supplement: S1 Data — (ZIP) [file pone.0298601.s001.zip › Data/地级市Z_F工作报告.zip!/║╙▒▒/╠╞╔╜╩╨/└┤╫╘╣1⁄2╓┌║┼í╛┬φ┐╦╩2╛▌═°í┐_2005.docx]

一、2004年政府工作回顾

刚刚过去的2004年，全市人民在市委的领导下，认真贯彻国家宏观调控政策和中央、省一系列决策部署，树立和落实科学发展观，围绕“朝着走在全省最前列加速前进”工作主题，积极进取，真抓实干，圆满完成了十二届人大二次会议确定的各项目标任务，改革与发展取得了新的成就。预计全市地区生产总值达到1606亿元，增长14.8 %，其中第一二三产业增加值分别增长5.8%、17.9%和13.7%;全部财政收入160.2亿元，增长45%。

——认真落实国家宏观调控政策，结构调整取得较大进展。坚持积极的、有作为的方针，以国家实施宏观调控政策为契机，努力消除经济发展中不稳定、不健康因素，推进经济结构调整，保持了经济平稳较快发展。采取坚决措施，对部分行业投资过热、乱占耕地及各类开发区进行了清理整顿，制止了一批不符合国家产业政策项目盲目上马。坚持从实际出发，把宏观调控政策措施与唐山实际紧密结合，适时适度、区别对待、有保有压，使固定资产投资保持了合理较快增长。全社会固定资产投资完成447亿元，增长42%。按照“上大压小、增高减低、扶优汰劣”的思路，下大力狠抓地方钢铁行业整治，对违规生产企业和落后生产设备进行了强力关停，促进了钢铁工业的健康发展。加强经济运行调节，缓解煤、电、油、运等瓶颈制约，为经济平稳较快发展提供了有力保障。全市规模以上工业企业增加值、利税分别增长26.5%和20%。

——扎实推进曹妃甸工业区开发建设，生产力布局向沿海推进迈出新步伐。曹妃甸工业区配套工程建设进展顺利。通港公路、青林公路全线贯通;矿石码头、围海造地以及供水、供电、通信等项目建设步伐加快;大型精品钢材基地、大秦线扩能分流、大型电厂、原油接卸码头等重大项目前期工作取得重要进展。京唐港功能进一步完善，港口运营达到2600万吨，增长25%。海港开发区和南堡开发区建设取得新成绩。沿海经济隆起带展现出巨大的发展前景。

——全面贯彻中央1号文件精神，农业农村经济健康发展。落实各项惠农政策，调减农业税率3个百分点，取消农业特产税，与上年相比农民税赋减轻49.6%;认真兑现种粮补贴、购买农机补贴和良种补贴，农民种粮积极性提高。全市粮食总产比上年增长9.2%。五大畜禽良繁体系不断完善，畜牧水产业占农业总产值的比重达到49%。认真做好高致病性禽流感防控工作，建立了市县乡三级动物防疫体系。乳业、瘦肉型猪、果菜、板栗、花生、水产品等农业龙型经济规模进一步发展壮大，产业化经营率达到53%。成功举办了首届唐山农产品展示交易会。文明生态村创建活动扎实推进，农村人居环境进一步改善。

——狠抓四项重点工作，经济发展活力和后劲进一步增强。 在项目与投资方面，以“四大兴市工程”为代表的重点项目建设取得突破性进展。曹妃甸工业区基础设施工程、大唐王滩发电厂一期工程、唐钢130万吨冷轧工程顺利推进，首钢200万吨钢联工程正式投产。全市50项省市重点项目完成投资120亿元，比上年翻一番。在深化改革方面，列入两年改制规划的229户国有独资、控股及参股企业，已有140户完成改制，其中市属企业38户。津西钢铁、开滦精煤上市成功。城镇职工基本养老、失业和医疗保险等社会保障制度改革继续深化。新一轮政府机构调整和职能整合顺利完成，国有资产监督管理体制进一步完善。行政服务中心功能不断增强。事业单位改革稳步推进。在发展民营经济方面，进一步完善政策引导机制，加大支持服务力度，促进民营经济健康发展。民营经济增加值占地区生产总值的比重达到52%，比上年提高4个百分点。在扩大开放方面，利用外资和对外贸易创历史最高水平。全市实际利用外资4亿美元，增长1倍;引进国内资金93亿元，增长31%。全年完成进出口总额18.2亿美元，其中出口8.9亿美元，分别增长80%和100.5%。外经工作取得新进展。成功举办了第七届唐山中国陶瓷博览会。

——城乡建设协调推进，城市化步伐加快。城市总体规划修编通过建设部评审，机场新区、曹妃甸工业区等17项重点区域规划基本完成。唐承高速、沿海高速开工建设，迁唐公路、平青大公路改造工程建成通车，村村通油路工程完成1759公里，城乡路网进一步完善。机场搬迁、国际会展中心、纪念碑广场改造等重点工程相继完工。商品房竣工110万平方米，经济适用住房竣工15万平方米;扩供煤气用户8554户，新增供热面积200万平方米。以争创国家卫生城为契机，狠抓城市基础设施、环境综合整治和生态建设，新建、改造、翻修城市干道28条，改造旧小区37个，城市绿化美化净化水平不断提高。加强生态环境保护和污染源治理，城市空气环境质量二级以上天数达到277天，比上年增加35天。南部采沉区生态建设项目荣获联合国“迪拜国际改善居住环境最佳范例奖”。县(市)城和小城镇建设水平明显提高，城镇面貌发生了新的变化。全市城市化率达到42%。

——“科教兴市”取得新成效，卫生、文化、体育、广电等各项事业全面发展。科技创新体系和平台建设取得新进展。“国家钢铁材料产业化基地”、“国家火炬计划唐山陶瓷材料产业基地”落户我市。组织实施科技攻关项目312项，科技进步对经济增长的贡献率达到55%。教育事业健康发展。义务教育水平得到巩固提高，“普九”工作通过省第三轮复查验收。调整撤并中小学197所，改造中小学校舍26.5万平方米。唐山外国语学校等一批教育设施竣工并投入使用。普及高中阶段教育取得积极进展，初中毕业生升学率达到74.3%。“四本多专”高等教育格局进一步完善。人才智力引进工作取得新成绩，新引进硕士生博士生 145名、各类紧缺人才3500名。加强公共卫生体系建设。市疾控中心主体工程完工，7个县(市)级疾控中心投入使用，乡镇卫生院全部上划县级管理。文化事业不断发展。文化基础设施建设得到加强，艺术创作、文化市场繁荣活跃，群众性文化活动丰富多彩。成功举办了第四届中国评剧艺术节和第三届唐山社区艺术节。全市继续保持低生育水平，计划生育率达到98%，人口自然增长率为4‰。广播电视节目更加贴近群众、贴近生活，数字电视、互联网用户普及程度不断提高。全民健身活动广泛开展。社区建设、社会福利、社会救济、优抚安置等民政事业取得新的进步。土地、矿产、海洋等自然资源得到有效保护和合理开发利用。民族宗教、外事侨务、旅游、防震减灾、气象、人防、档案等各项工作也都取得了新成绩。

——城乡人民群众实际收入增加，生活质量进一步改善。全市城镇居民人均可支配收入达到8902元，农民人均纯收入4083元，分别增长10.3%和7.7%，是近年来增幅最高的一年。城乡居民消费水平进一步提升，全社会消费品零售总额达到359亿元，比上年增长17%。就业和再就业工作取得新成效。全年城镇新增就业人数4.3万人，2.7万名下岗职工实现了再就业;城镇登记失业率为4.1%。国有企业下岗职工基本生活保障率达到100%，离退休人员基本养老金和失业人员失业保险金按时足额发放。制定并实施了城镇最低收入家庭住房保障暂行规定，对享受最低生活保障线的住房特困户、无房户实行了租赁住房补贴和租金核减。城市居民低保标准由每人每月199元提高到205元，建立了全市统一的农村低保制度，农村低保标准每人每年800元，城乡困难群众基本生活得到有效保障。市政府为群众办的23件实事全部完成。

——社会主义精神文明建设迈出新步伐，民主法制建设不断加强。以创“五城”为载体，深入开展公民道德教育实践活动和群众性精神文明创建活动。在巩固扩大全国双拥模范城和国家园林城创建成果、推进全国文明城和国家环保模范城创建工作的基础上，加大了创建全国卫生城活动的推动力度，开展了“创卫百日会战”，城乡环境卫生和文明水平进一步提高。坚持并完善了向人大报告工作、向政协通报情况制度，自觉接受人大法律监督和工作监督，重视发挥政协的政治协商、民主监督和参政议政作用，提高了政府决策科学化、民主化水平。承办人大代表意见建议145 件，政协委员提案578件，全部按时办结;规划、劳动、林业等政府组成部门负责人向市人大常委会述职并接受评议。认真贯彻行政许可法，依法清理各级各类行政许可事项1367项;向人大提交地方性法规草案3部，出台地方政府行政规章7部;行政执法责任制进一步落实;普法教育、基层民主政治建设不断深入，政府工作法制化水平进一步提高。认真做好人民群众来信来访工作，努力为群众排忧解难。廉政建设不断加强，“阳光行动”、行风评议扎实推进，反腐败斗争取得阶段性成果。高度重视并切实抓好安全生产，加强市场经济秩序整顿。重视开展隐蔽战线斗争，加强与“法轮功”邪教组织的斗争，严厉打击各种刑事和经济犯罪，积极开展“诚信平安唐山”活动，强化社会治安综合治理，维护了全市政治安定和社会稳定。全民国防教育进一步深入，民兵预备役工作得到加强。

各位代表!过去的一年，我市国民经济和社会发展取得了令人鼓舞的成绩，朝着实现“走在全省最前列”的奋斗目标跨出了重要一步。这是在市委的领导下，全市人民团结奋斗的结果，是市人大、市政协监督帮助和全市各界人士关心支持的结果。在此，我代表市政府，向全市人民致以崇高的敬意!向驻唐部队指战员、武警官兵、政法公安干警以及关心支持唐山建设的各界人士和朋友，表示衷心的感谢!

各位代表，面对我市当前的大好形势，我们也清醒地看到，经济和社会生活中还存在不少矛盾和问题：经济增长方式粗放，经济结构调整特别是钢铁工业调整任务艰巨;煤、电、油、运等“瓶颈”制约仍未得到有效缓解;经济增长与资源、环境之间的矛盾比较突出;经济体制改革特别是国有企业改革有待进一步深化;农民增收的基础还不稳固;就业和再就业压力依然很大，部分低收入家庭生活还比较困难;政府机关思想和工作作风还不适应新形势新任务要求，尤其是在倾听群众呼声、解决群众实际问题方面仍有较大差距;个别地方和部门仍然存在消极腐败、不正之风等问题。对这些问题，我们一定引起高度重视，在今后工作中认真加以解决。 二、2005年工作总体思路、奋斗目标和任务

2005年是我市全面完成“十五”计划，加快全面建设小康社会，朝着走在全省最前列目标前进的关键一年。我们必须居安思危，增强忧患意识和责任意识，倍加珍惜难得的发展机遇、倍加珍惜良好的发展局面、倍加珍惜来之不易的发展环境，再接再厉，乘势而上，努力把我市改革开放和现代化建设推向新阶段。今年我市国民经济和社会发展的总体思路是：深入贯彻邓小平理论和“三个代表”重要思想，认真落实党的十六届四中全会、省委六届六次全会和市委七届六次全会精神，用科学的发展观统领经济社会发展全局，坚持以经济建设为中心，以积极的、有作为的姿态落实宏观调控政策，突出结构调整，转变增长方式，认真解决“三农”问题，继续狠抓“项目建设、国企改革、民营经济、对外开放”四项重点工作，坚持统筹兼顾，大力发展社会事业，全面加强社会主义精神文明和民主法制建设，维护社会稳定，构建文明和谐唐山，坚持以人为本，努力提高人民群众的生活水平和质量，推动经济社会更快更好发展，为实现走在全省最前列的目标不懈奋斗。全市经济社会发展的主要预期目标是：全市地区生产总值增长13%左右;全部财政收入增长13%以上，全社会固定资产投资增长18%，实际利用外资增长12.5%，进出口总额增长20%;全社会消费品零售总额增长13%;城镇登记失业率控制在4.5%左右，城镇居民人均可支配收入和农民人均纯收入分别增长7%和5%。上述预期目标是在认真分析形势，充分考虑各方面因素的基础上提出的，是加快我市发展和不断提高人民生活水平的需要，我们必须用最大的决心努力实现。

二、贯彻上述总体要求，实现上述工作目标，需要统筹抓好以下六项重点工作：

(一)深入贯彻宏观调控政策，加快经济结构战略性调整和经济增长方式转变

巩固和扩大宏观调控成果。继续坚持积极的、有作为的方针，抓好中央各项政策措施的落实。着力优化投资结构，认真贯彻区别对待、有保有压的原则，加大对经济社会发展薄弱环节的支持力度，支持农业基础设施、缓解瓶颈制约重大项目的建设，增加对基础教育、公共卫生体系、生态建设和环境保护等方面的投入;合理引导社会资金投向，对符合国家产业政策和我市结构调整方向的投资，予以大力支持;严格控制盲目投资，防止低水平重复建设。坚持依法、节约、集约用地，盘活存量土地，努力增加建设用地有效供给。加强和改善对经济运行的调节，搞好重要原材料供需衔接，增加电力、煤炭等重点生产要素供应，缓解煤电油运紧张状况。加强市场物价监管，维护市场秩序，保持物价稳定。

加快生产力布局向沿海推进。深入实施以港兴市战略，充分发挥区位、港口和海洋资源优势，以曹妃甸工业区为核心，以海港开发区、南堡开发区为支撑，统筹规划，合理布局，整体推进，加快沿海一线开发建设步伐。以发展大码头、大钢铁、大电厂、大石化为目标，全面推进曹妃甸工业区基础设施建设，力争年内基本达到配套条件，为项目进区奠定基础。加快曹妃甸精品钢铁基地和矿石、煤炭、石油、天然气四大码头建设，力争矿石码头年内建成，煤炭、石油、天然气码头具备开工条件，钢铁基地完成前期工作。狠抓京唐港和海港开发区建设，完善港口功能，提升港口运力;积极推进大唐发电、北焦化等大项目建设。加快南堡开发区发展步伐，重点实施三友集团烧碱、PVC和差别化粘胶纤维等项目。依托港口和沿海“三区”，大力发展海洋经济和临港产业，加速形成沿海经济隆起带。

加大工业经济结构调整力度。全市规模以上工业企业增加值、利税分别增长20%以上。改造提升传统产业。围绕打造精品钢材、优质建材、基础能源、机械制造和化工五大产业基地，积极运用高新技术和先进适用技术，加快传统产业技术改造步伐。以建设钢铁强市为目标，按照“品种、质量、整合”的思路，坚持扶优汰劣，积极推进不同所有制钢铁企业走联合发展之路，促进集约化经营;加快调整钢铁产品结构，积极发展钢铁工业深加工，延长产业链，巩固扩大市场占有率。充分发挥钢铁协会作用，促进资源整合和行业自律。加快高新技术产业发展。着力发展电子信息、机电一体化、生物工程、新材料等高新技术产业群体，积极培育新兴产业，重点抓好50个高新技术产业化和技术创新项目。加快高新技术开发区建设步伐。大力发展循环经济。坚持开发和节约并举、把节约放在首位的方针，逐步构建节约型产业结构和节约型社会。实行高能耗、高物耗设备和产品淘汰制度，重点抓好冶金、建材、造纸等行业节能节水技术改造。大力推行清洁生产，积极推广矢量变频节电等新工艺新技术，重点耗能工业企业万元产值综合能耗降低5%。积极推进“三废”资源化，提高“三废”回收循环利用水平。

大力发展服务业。面向城乡居民消费和扩大就业，按照扩大总量、优化结构、拓展领域、提高层次的思路，改造提升传统服务业，积极发展现代服务业。坚持发展服务业与壮大区街经济有机结合，把服务业作为区街经济发展的重点，发挥优势，完善政策措施，推动服务业上规模、上档次、上水平。积极推进社区服务社会化、产业化、市场化。制定并实施唐山市商业发展规划，鼓励城区污染企业和亏困企业实施“退二进三”，开发建设服务业项目。在发展壮大传统服务业的同时，积极推行连锁经营、配送、电子商务等现代物流业态;加快发展房地产、金融保险、中介咨询等新兴服务业;大力发展信息产业，加快国民经济信息化进程;整合旅游资源，搞好规划，完善基础设施，打造旅游品牌，把旅游业做大做强。

(二)切实解决好“三农”问题，全面发展农业农村经济

加大对农业的扶持力度。全面落实中央各项惠农政策，农业税税率再降低2个百分点，继续实行对种粮农民的直接补贴，增加良种补贴和农机具购置补贴，调动和保护农民生产积极性。加大财政支农力度，实行工业反哺农业、城市支持农村的方针，开辟新的支农资金渠道，形成农业投入稳定增长机制。搞好农业技术推广，积极开展实用技术培训，加强农资市场价格监管，进一步完善支农服务体系。

调整优化农业结构。高度重视粮食生产，保护和提高粮食综合生产能力。坚持以市场为导向，按照“把龙头做强、基地做优、产品做精、品牌做响”的思路，大力发展精品农业、特色农业、绿色农业，积极推进农业标准化生产和外向化经营，打造农业知名品牌。加快发展畜牧水产业，加强“五大良繁体系”和动物防疫体系、农产品质量检测体系建设，推动畜牧水产业向专业化、规模化、工厂化发展，畜牧水产业占农业增加值比重达到50%。

加快推进农业产业化经营。继续壮大乳业、瘦肉型猪、果菜、板栗、花生、水产品六大龙型经济，积极培育肉牛、肉羊、食用菌、甘薯、饲草、中药材等产业。做大做强龙头企业，重点扶持一批有市场前景的企业加快发展，鼓励一批知名企业扩大规模。积极发展农村专业合作组织。在完善乳业、板栗、食用菌行业协会的同时，组建瘦肉型猪、蔬菜、水产品等行业协会，积极培育专业合作社和经纪人队伍。全市农业产业化经营率达到55%。

加强农业基础设施建设。抓好水利工程项目建设，发展节水灌溉面积16万亩。继续推进集雨水窖和平原水库建设，充分利用好雨洪资源。启动农村饮水安全项目，切实解决好人畜饮水困难问题。继续推进农村文明生态村建设，按照每年10%的计划要求，年内再完成650个重点村创建任务。

进一步深化农村改革。坚持产业化、工业化、城镇化关联互动的思路，加快县域经济由农业主导型向工业主导型转变。大力发展农村二三产业，积极推进农村工业化进程，加速农村剩余劳动力向非农产业和城镇转移，拓宽农民增收渠道。继续推进农村税费改革，积极稳妥地搞好乡镇机构改革、农村义务教育体制改革和县乡公共财政制度改革，进一步减轻农民负担。落实粮食购销市场化改革措施，加快推进国有粮食购销企业转变经营机制。推进农村信用社改革，改善农村金融服务。依法稳定农村土地承包关系，深化征地制度改革。

(三)做深做实四项重点经济工作，增强经济发展动力、活力和后劲

继续狠抓项目建设。围绕结构调整这条主线，突出重点项目、统筹中小项目，增强项目建设对经济社会发展的支撑力和拉动力。抓好重点项目建设，全年安排重点项目80个，年度计划完成投资200亿元，力争240亿元。加快35个续建项目建设，确保15个项目完工;抓好45个新开和争取开工项目准备工作，争取早日开工。进一步创新项目工作思路，坚持经济效益、社会效益、扩大就业并重，加大项目谋划和前期工作力度，做到一二三产项目齐抓，大中小项目并举。加强项目宏观管理，正确引导资金投向，调动和保护投资者积极性。搞好银企合作，加大金融对项目建设的支持力度。

全面深化各项改革。国有企业改革，坚持以“两增、两减、两分”为主线，紧紧围绕产权制度改革、职工身份转换和出资人到位等关键问题，规范推进国有企业改革。认真落实国有企业改革实施方案和规划，对列入两年改制规划尚未完成改制的89户国有企业，年内确保全部完成改制任务;继续对改制不到位、不彻底的企业实施二次改制。加快现代企业制度建设，健全公司法人治理结构，完善激励约束机制，全面实行国有企业经营业绩考核制度。大力发展混合所有制经济，推动国有、集体、民营、外资在更大范围内流动重组。进一步健全完善国有资产管理和监督机制，完善国有资产评价、监管体系，防止国有资产流失，维护出资人和职工合法权益。投资体制和财政体制改革，完善固定资产项目投资备案和核准实施办法，建立健全投资监管体系，确立企业投资主体地位。建立政府及国有企业投资决策责任制，规范政府投资范围和行为。按照建立公共财政要求，深化财政预算体制改革。依法加强财税征管，合理调整收入分配结构。建立支出效益评价、考核监督、责任追究制度，严肃财经纪律，提高财政资金使用效益。大力开展增收节支，坚持厉行节约，反对铺张浪费。各项配套改革，进一步深化城镇养老、失业、医疗、工伤保险制度改革，完善社会保障体系。加快发展资本、土地、技术、劳动力、产权等要素市场。稳步推进机关事业单位改革。加强机构编制管理，确保全市财政供养人员控制目标和精简任务的完成。

努力扩大双向开放。提高利用外资规模质量。抓住我国加入WTO进入“后过渡期”的机遇，优化投资环境，扩大开放领域，提高招商引资实效。鼓励外商投资高新技术产业、先进制造业、现代服务业、现代农业和环保产业，突出抓好一批利用外资重点项目。搞好开发区和各类园区建设，充分发挥其招商引资平台和示范带动效应。办好第八届唐山中国陶瓷博览会，活跃对外开放全局。努力扩大对外贸易。继续实施大经贸、以质取胜和市场多元化战略，加快转变对外贸易增长方式，调整优化结构，积极推动钢铁、水泥、机电、陶瓷和纺织等具有比较优势的产品出口，培植大宗商品和创汇大户;发挥港口优势，积极发展进口和加工贸易。加大对出口企业的支持力度，引导和保护好出口企业的积极性。搞好对外经济技术合作。大力实施“走出去”战略，鼓励更多的企业到海外投资办厂，扩大工程承包和劳务合作。进一步扩大对内开放。抓住国家启动京津冀都市圈区域规划的有利时机，加强与京津、东北、西部地区的联合与协作，实现优势互补、产业互动。

推动民营经济加快发展。坚持把发展民营经济作为加快经济发展的重大举措来抓，认真贯彻落实各项鼓励支持民营经济发展的政策措施，切实解决民营经济发展中的困难和问题，推动民营经济总量扩张、质量提高。全市民营经济占地区生产总值的比重达到54%以上。拓展民营经济发展空间。积极引导民营资本进入法律法规未禁入的基础设施、公用事业及其它行业和领域，鼓励民营资本通过收购、兼并、投资入股等形式参与国有企业改制、改组和改造。引导民营企业提高产业层次、产品档次和企业素质。及时向民营企业传导政策信息和项目信息，帮助民营企业正确把握投资方向，自觉执行国家产业政策和技术、质量、环保、安全等市场准入标准，积极推进制度创新、科技创新和管理创新，把企业做大做强。改进对民营经济服务和监管。完善中小企业信用担保体系、技术创新服务体系和创业辅导体系，在资金、技术、信息、法律等方面为民营企业提供优质服务。加强对民营经济管理，使民营企业自觉依法经营、诚实守信。依法维护民营企业及职工的合法权益。

(四)统筹推进各项社会事业，实现全面协调发展

加快城乡建设步伐。围绕建设现代化沿海大城市，深入实施城市化战略，提高城市现代化水平。全市城市化率达到43.5%。搞好城市规划设计。按照生产力要素向城镇聚集、生产力布局向沿海推进的思路，进一步做好南部沿海发展规划，加快编制机场新区、南湖生态区等重点地区控制性详规，合理规划县城和小城镇，科学引导和调控城市建设。加快城市基础设施建设。启动机场新区开发建设，力争经过几年努力，将机场新区打造成具有国际水准的现代化新城区。制定城区道路交通规划，完善城市路网，改善交通状况。年内新建、改造、整治城市道路43条，谋划实施市中心区至丰润区汽车专用快速通道。抓好市区垃圾填埋厂和市中心区三期供水工程、城区供水管网改造等一批市政公用设施项目建设;统筹商品房开发和经济适用住房建设，完善经济适用住房出售、管理办法，促进房地产市场健康有序发展。深化城市建设管理体制改革。按照“两级政府、三级管理、四级落实”的要求，强化街道在城市管理中的基础地位，推进城市管理重心下移。加强市容市貌综合整治，提高城市净化绿化美化亮化水平。引入市场竞争机制，加快公用企(事)业单位改革步伐，确保取得实质性进展。积极推动城镇发展。抓好县城和城镇以路水电讯为重点的基础设施建设，增强辐射带动功能，为聚集产业、吸纳人口创造条件。完善城乡交通体系，加快唐承、沿海高速和国省干线公路建设。全市基本实现村村通油路。

推进科技教育发展。加速科技进步。完善科技人才、科技合作、科技发展与公共服务三个平台，不断优化科技发展环境。紧紧围绕经济发展和结构调整，加快科技创新步伐，着力实施海水淡化科技示范工程等28项科技攻关项目。抓好国家制造业信息化工程示范城市和国家钢铁材料产业化基地、国家火炬计划陶瓷材料产业基地、唐山民营科技特色产业基地建设。加快发展教育事业。巩固提高“普九”水平，整合教育资源，进一步改善义务教育办学条件。调整撤并中小学120所，全面完成现有22万平方米危旧校舍改造任务。落实“以县为主”的农村教育管理体制，保障教育经费需要。加快普及高中阶段教育，扩大优势高中规模，抓好唐山一中迁建。初中毕业生升学率达到78.2%。搞好高等院校规划建设，加快高等教育发展步伐。全面实施素质教育，提高教学质量。大力发展职业教育和继续教育，积极发展民办教育。切实加强教师队伍建设。认真治理教育乱收费。继续实施“人才强市”战略，紧紧围绕经济社会发展需要，抓好人才培养、引进和使用工作，创新人才政策，优化人才环境，提高人才效益。

大力发展卫生、文化等各项社会事业。卫生事业，推进城乡医疗卫生机构改革，加强公共卫生体系建设。抓好市急救中心、市县两级疾病预防控制中心和传染病医院建设，认真做好传染病防治工作，提高公共卫生保障能力。积极发展社区卫生服务。加强农村医疗卫生基础设施建设，实施乡镇卫生院标准化建设三年规划工程。继续抓好以大病统筹为主的新型农村合作医疗和农村医疗保障制度改革。深入开展爱国卫生运动。文化事业，制定实施文化强市建设规划，加快文化产业发展。深化文化管理体制改革，整合文化资源，推进文化单位转机建制，打造文化艺术精品。加强文化基础设施建设。广泛开展社区、农村、企业、校园、家庭等群众性文化娱乐活动，大力弘扬冀东地域文化，丰富群众文化生活。办好首届中国唐山国际皮影艺术展演。体育事业，加快实施全民健身计划，抓好全民健身工程建设，广泛开展群众性体育活动，增强人民身体素质。加速培育体育人才，提高体育竞技水平。精心筹备2007年第十二届省运会。广电事业，加快广播电视数字化、网络化、产业化建设，提高节目质量和水平。全面贯彻落实党的民族宗教政策，依法管理宗教事务，促进民族团结。关心支持妇女儿童、慈善、老龄和残疾人事业，进一步做好外事侨务、人防、防震减灾、档案、气象等工作。

推进可持续发展。统筹做好人口、资源、环境等各方面工作，推动经济和社会走上资源永续利用、生活安康富裕、生态环境良好的文明健康发展轨道。做好计划生育工作。继续稳定低生育水平，促进优生优育，提高人口素质。全市人口自然增长率控制在5‰以内。搞好自然资源合理开发和利用。依法保护水、土地、矿产、海洋等资源。加强矿产资源的规划、开发和管理，严禁滥采乱挖和资源浪费，提高综合利用效率。特别要加强水资源统一管理和科学配置，建设节水型社会。以建设林业生态体系和林业产业体系为重点，大力植树造林和封山育林，打造“绿色唐山”。加强对大气、水、垃圾、机动车尾气和噪音等污染防治，全面落实污染物总量控制计划。抓好冶金、水泥、造纸、电力、陶瓷等重点行业污染源治理，削减污染物排放总量。认真落实项目环境评价制度，防止破坏生态环境的项目盲目上马。加强对沿海湿地、近海海域及海洋生物的保护。

(五)坚持以民为本，切实改善人民生活

努力增加城乡居民收入。在提高经济效益的基础上，努力提高城镇居民特别是低收入者收入水平。通过加快城镇化、调整农业结构和劳动力结构、发展农业产业化等多种途径，千方百计为农民增收广开门路。加强劳动监察，落实城镇职工最低工资标准。继续组织实施好为群众办实事工程，妥善解决好水库移民、失地农民、城镇房屋拆迁补偿、农村土地征用补偿等事关群众切身利益的问题。

千方百计做好就业和再就业工作。继续把就业再就业放在突出位置，坚持在发展中解决就业问题，逐步建立有利于扩大就业的经济结构和增长模式。注重发展就业容量大的服务业、中小企业、农副产品加工业和劳动密集型产业，形成更多的就业增长点。用足用好财政补贴、小额贷款、免费培训、税费减免等促进就业再就业各项政策措施，确保落到实处。妥善做好下岗职工出中心向失业保险并轨工作。完善公共就业服务体系。加强劳动力市场建设，建立劳动就业监测预警体系。认真贯彻《劳动保障监察条例》，规范劳动力市场秩序和企业用工行为，保障劳动者合法权益。统筹做好大中专毕业生、军转人员等各方面的就业工作。

健全完善社会保障体系。扩大城镇基本养老、基本医疗和失业保险覆盖面，依法加强社保基金征缴，推行职工工伤保险和生育保险。推进企业退休人员街道社区社会化管理服务工作，做好“三条保障线”的衔接，确保国有企业下岗职工基本生活保障金和离退休人员基本养老金按时足额发放。高度重视社会弱势群体，提高城镇最低生活保障线标准，完善城镇最低收入家庭医疗救助、教育救助、法律援助和住房保障制度，加快发展社会福利事业。

(六)加强精神文明和民主法制建设，构建文明和谐社会

加强社会主义精神文明建设。坚持用毛泽东思想、邓小平理论和“三个代表”重要思想占领思想阵地，进一步提高思想政治工作水平。在全社会大力弘扬以爱国主义为核心的民族精神和以改革创新为核心的时代精神，大力弘扬抗震精神，使全市人民始终保持昂扬向上的精神状态。认真贯彻《公民道德建设实施纲要》，加强公民尤其是未成年人思想道德建设，深入开展公民道德实践活动，巩固和发扬团结互助、平等友爱、共同前进的社会主义新型人际关系。以创“五城”为载体，广泛开展群众性精神文明创建活动，提升社会文明程度。深入开展国防教育，加强军政军民团结，支持驻唐部队和民兵预备役建设。

推进社会主义民主法制建设。按照科学执政、民主执政、依法执政的要求，大力发展社会主义民主政治。自觉接受人民代表大会及其常委会的法律监督和工作监督，自觉接受人民政协的民主监督、政治协商和参政议政，认真办理人大代表、政协委员的批评、意见、建议和提案。积极听取民主党派、工商联、无党派人士及各人民团体意见。进一步完善公众参与、专家讨论、政府决策相结合的决策机制和重要决策失误责任追究制。建立健全社情民意反映制度、重大事项社会公示、听证、质询制度。加强基层政权建设和群众性自治组织建设，落实好各项公开制度，保证群众依法行使民主权利。大力推进依法治市。继续深入开展“四五”普法宣传教育，提高全民特别是公职人员的法制观念和法律素质;加强执法制度建设，落实好国务院《依法行政实施纲要》，推进政府工作法制化。

维护社会稳定和谐。正确处理改革、发展、稳定的关系，注重和维护社会公平。加强社会利益协调，把最广大人民群众的根本利益作为制定政策、开展工作的出发点和落脚点，兼顾不同方面群众的利益，特殊关爱弱势群体，保证人民群众都能享有改革发展的成果，促进社会公平。切实做好行政复议、仲裁、信访、民调等各项工作，及时化解各种社会矛盾。全面落实安全第一、预防为主的方针，高度重视并坚持不懈地抓好安全生产，坚决防止重特大安全事故的发生，切实保障人民群众生命财产安全。建立健全社会预警体系和应急反应机制，预防和妥善处置群体性事件，提高保障公共安全和处理突发事件的能力。全面落实社会治安综合治理各项措施，依法严厉打击各种刑事犯罪和经济犯罪，高度重视隐蔽战线斗争，加强同“法轮功”等邪教组织的斗争，深入开展“诚信平安唐山”活动，创造安全稳定的社会环境。 三、切实加强政府自身建设

新形势、新任务，对政府工作提出了新的更高的要求。我们要牢记立党为公、执政为民的宗旨，大力推进政府自身建设。

转变职能，优化发展环境。深化行政体制改革，推进政企、政事、政资分开，在继续履行好经济调节和市场监管职能的同时，更加注重强化社会管理和公共服务职能，努力建设服务型政府。全面落实优化发展环境的各项政策措施，着力解决思想观念、办事效率、管理公开公正、司法保障及社会诚信等方面的突出问题。严格执行《行政许可法》，进一步改革行政审批制度，简化审批程序，规范行政行为，推进电子政务，提高行政效率和服务水平。健全社会信用体系，打造诚信唐山。建立健全发展环境评价考核机制，对行政不作为、乱作为的人和事，坚决查处，决不姑息，努力营造创业宽松、社会文明、人居安全和低生产成本、低交易成本、低行政成本的发展环境。

依法行政，建设法治政府。以建设法治政府为目标，积极推进政府管理理念、管理机制和管理方式的创新，坚持依法行政，提高政府工作人员法制观念，增强全面落实科学发展观的能力，增强按市场经济规律办事的能力，增强运用法律手段调节经济运行的能力，提高政府工作规范化、法制化水平。进一步健全完善重大行政行为事前合法性审查制度和行政工作人员行政违法行为责任追究制度，强化规范性文件备案审查制度，完善行政监督机制，坚决纠正不规范行政行为。

执政为民，大兴求真务实之风。进一步强化民本意识，牢固树立正确的政绩观，真正做到重大决策依民而定，工作措施应民而出，安危冷暖唯民而系。坚持深入基层和实践，注意倾听基层和群众呼声，体察民情，了解民意，下大力解决好人民群众关注的热点难点问题，尽心尽力为群众排忧解难。发扬求真务实的作风，脚踏实地，埋头苦干，不图虚名，把主要精力集中到抓重点、抓大事、抓主要矛盾上来，用在抓工作落实上来，把各项工作扎扎实实推向前进。

标本兼治，扎实推进廉政建设。牢记“两个务必”，始终保持谦虚、谨慎、不骄不躁的作风，保持艰苦奋斗的作风，常修为政之德、常思贪欲之害、常怀律己之心，真正做到勤政、务实、廉洁、高效。认真落实党风廉政建设责任制，坚持标本兼治、综合治理、惩防并举、注重预防的方针，建立健全教育、制度、监督并重的惩治和预防腐败体系。加强理想信念教育、廉洁从政教育和警示教育，增强各级公务员拒腐防变的能力。进一步健全完善并严格执行政务公开、行政审批、政府采购等各项工作制度，加强对经济活动特别是政府开支的审计监督。继续推进民主评议和“阳光行动”，坚决纠正群众反映强烈的部门和行业不正之风。坚决查处违法违纪案件，严厉惩治腐败分子，取信于民。

各位代表，今年的任务光荣而艰巨。让我们在中共河北省委、省政府和市委的统一领导下，抓住机遇，开拓进取，同心同德，扎实工作，为圆满完成全年目标任务、实现走在全省最前列的目标而不懈奋斗!
